# Time‐Quantile and Frequency Response of Green Growth to Energy Vulnerability, Energy Uncertainty, and Geopolitical Risks

**DOI:** 10.1002/gch2.202400225

**Published:** 2024-10-13

**Authors:** Emmanuel Uche

**Affiliations:** ^1^ School of Economics College of Business & Economics University of Johannesburg Johannesburg Gauteng 234 South Africa; ^2^ Middle East University Amman Airport Rd. Amman 11831 Jordan

**Keywords:** energy efficiency, energy uncertainty, energy vulnerability, geopolitical risks, green growth

## Abstract

The study explains the time‐quantile‐frequency adjustments of green growth to energy vulnerability, energy uncertainties, and geopolitical risks (GPR) in the United States (US). Novel insights with notable policy implications emerged following the empirical analysis of monthly data spanning 2000 m1–2022 m12. The study implemented the Wavelet Quantile Correlation (WQC), Wavelet Quantile Granger Causality, and the Rolling Windows Wavelet Quantile Granger Causality to understand the dynamics among the variables. Evidence from WQC divulged time‐specific positive and negative interactions between green growth and its determinants. Specifically, energy vulnerability dampened green growth more profoundly in the immediate and medium terms. However, in the long term, green growth prospers amidst energy vulnerability. This outcome reflects some policy effectiveness that reduced the negative effects of energy vulnerability for green growth. The effects of energy uncertainties are similar to that of energy vulnerability, with more profound damaging effects in the lower medium horizon of the distributions. GPR dampened green growth in the short run and enhanced it in the medium term, but it reduced green growth more profoundly in the long run. The pleasant effects of energy efficiency and digitalization are observed mostly in the long run, with notable green growth‐reducing effects mainly in the short run.

## Graphical abstract

This study explores the time‐quantile‐frequency adjustment path of green growth to energy vulnerability, uncertainties, and geopolitical risks, focusing on the US economy. The analysis uncovers that these factors dampen green growth more profoundly in the immediate and medium terms. However, in the long term, green growth prospers amidst energy vulnerability. Further insights highlight the pollution‐reducing effects of energy efficiency and digitalization.

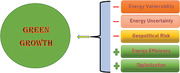

## 1 Introduction

Environmental sustainability administrators are mandated to provide insights into the best strategies for curtailing the vagaries of climate change. Delivering such streamlined insights becomes more daunting when curtain‐edge empirical elaborations are in short supply. The devastating effects of climate change and the urgent need to keep the environmental ecosystem at its optimal point of 1.5 °C based on the Paris Agreement have also made their task more daunting. With this realization, the United Nations (UN) and other relevant stakeholders have made significant inputs, including the Sustainable Development Goals (SDGs) initiatives, to ensure a world optimally resilient to climatic changes.^[^ ^1^, ^2^ ^]^ Understandably, many factors, including energy consumption and geopolitical tension, contribute to subsisting global warming.^[^ ^3^, ^4^, ^5^ ^]^ Such factors can potentially undermine the green growth initiatives canvassed by the UN and other international agencies. Hence, precise narrations and understanding of factors impacting green growth positively or negatively are of policy relevance. Such empirical narratives are imperative in designing environmental management policies to reduce climate change and its externalities.

The contributions of energy and its related factors to global warming cannot be overemphasized, given its highlighted adverse effects. It is documented that energy contributes ≈56% to climate change.^[^ ^5^, ^6^, ^7^, ^8^, ^9^, ^10^, ^11^ ^]^ However, what is not adequately addressed is the contributions of energy vulnerability and uncertainties to green growth initiatives of global leading economies. Green growth entails achieving higher productivity (economic growth) with minor damage to the environmental ecosystem.^[^ ^12^, ^13^ ^]^ That process decouples the economy from carbon and environmental pollution through efficient allocation and utilization of available resources.^[^ ^14^ ^]^ According to the understanding of the United Nations Environment Programme,^[^ ^15^ ^]^ the World Bank,^[^ ^16^ ^]^ and the Organization for Economic Cooperation and Development,^[^ ^17^ ^]^ green growth entails optimal utilization of available resources that do not compromise the environment. Deducting from the submissions of Merino–Saum,^[^ ^18^ ^]^ Telukdarie et al.^[^ ^19^ ^]^ argue that green growth gravitates toward four fundamental concepts: ecological scarcity, human well‐being, equity, and environmental vulnerability. Overall, green growth is a comprehensive barometer of economic growth that entails optimal resource harmonization and sustainability.

Hence, we emphasized that a clear understanding of the implications of factors like energy vulnerability, energy uncertainty, and geopolitical risk factors on green growth is fundamental for preventing rather than combating climate change and its externalities. However, prior studies have vastly explored the contributions of green growth and green energies to sustainable development.^[^ ^20^, ^21^, ^22^ ^]^ In comparison, only a few studies have explored the relevant factors facilitating green growth. These few studies dwelled on the effects of structural change on green growth,^[^ ^23^ ^]^ economic policy uncertainty – green growth nexus,^[^ ^14^, ^24^ ^]^ financial inclusion/development–green growth nexus,^[^ ^25^, ^26^, ^27^ ^]^ green innovation–green growth nexus,^[^ ^26^ ^]^ and geopolitical risk–green growth nexus.^[^ ^28^, ^29^, ^30^ ^]^ These studies provided the necessary incentives to probe other factors influencing green growth, given their fragmented submissions and the apparent negligence of the implications of critical factors like energy vulnerability and energy uncertainty. Hence, this study aims to verify the contributions of energy vulnerability and energy uncertainty to green growth in the United States (US) since it is the second‐largest energy‐consuming economy globally.^[^ ^31^ ^]^

**Figure 1:**
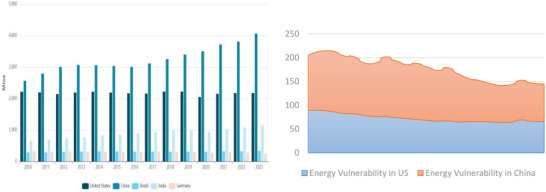
a) Energy consumption trends ‐ 2010–2023. b) Energy vulnerability trends ‐ 2010–2023.

As earlier highlighted and evidence emanating from **Figure** **1**a, the US is the second largest energy consumer after China. Evidence from global energy trends indicates that while China consumed 4060 Mtoe of energy in 2023, the US consumed 2172 Mtoe in the same year.^[^^31]^ Comparatively, the US, followed by Norway, Canada, and Australia, is more resilient to global energy pressure than China (Figure 1b).^[^ ^32^ ^]^ The US's least vulnerability to energy pressure is enhanced by high energy self‐sufficiency with the least dependence on external sources.^[32^^]^ This contrasts with China and other emerging economies still grappling with high energy vulnerability due to rapid industrialization, urbanization, and large dependence on traditional energies. Given this scenario, the critical question is whether the US's resilience to energy vulnerability is beneficial for green growth. Can the US economy achieve the expected green growth targets by remaining resilient to global energy pressures? These are critical questions that this study seeks to answer with the aim of disseminating policy insights for both the US and other global economies grappling with energy vulnerability. Unfortunately, prior studies have not considered this perspective, incentivizing the current investigation.

This study contrasts with prior studies by exposing green growth's time‐quantile and frequency response to energy vulnerability, uncertainty, and geopolitical risks. On this basis, this study is probably the first to simultaneously apply both the wavelet quantile correlation (WQC), the wavelet quantile Granger causality (WQGC), and the rolling windows wavelet quantile Granger causality (RWWQGC) in the context of green growth determinants in the US. This step allows for holistic and elaborate articulations of the dynamic and intricate associations among energy vulnerability, uncertainty, geopolitical risks, and green growth. Because of its focus on the world's largest economy, understanding such dynamics would allow for easy planning and policy adjustments toward sustainable environments. It will equip the US environmental administrators to be forward‐looking and imbibe proactive climate change resilient protocols rather than reactive processes. The outcomes based on such innovative analytical frameworks are a shift from conventional protocols that produce limited insights on evolving comprehensive climate change resilient strategies. Such narratives are lacking in extant studies that mainly explore the green growth – sustainable development nexus. This study would highlight where the US environmental policies are lacking and areas to consolidate for overall environmental vitality. It will open up new policy insights and spotlight areas where existing policies perform credibly or otherwise for policy moderation. In contrast, few others considered the implications of isolated factors on green growth, limiting policy insights into the main factors affecting green growth.

Other study components include the review of related literature in Section 2 and the relevant data, modeling, and applicable techniques in Section 3. The penultimate section elucidates the empirical analysis and discussions, while the study is concluded in Section 5 by highlighting the critical policy implications of the findings.

## 2 Literature Review

This component of the study explores the theoretical connections between energy vulnerability and green growth. Green growth conceptualization imbibes a series of economic activities that leave minimal damage to the environment.^[^ ^24^ ^]^ The green growth theory emphasizes a harmonious relationship between economic growth, natural resource consumption, and the environmental ecosystem.^[^ ^33^ ^]^ This process entails the integration of environmental concerns with economic growth by ensuring mutual relationships among them.^[^ ^34^ ^]^ Green growth theory aligns with the ecological modernization principles of Janicke,^[^ ^35^ ^]^ which seek growth expansion that does not compromise environmental progress. In the course of theoretical developments and understanding, many scholars have explored different drivers of green growth. Notable is the scant attention paid to factors like energy vulnerability and energy uncertainty. Understanding all relevant factors that may promote or impede green growth will make room for effective planning and responses to uphold a healthy environment.

In a bid to unravel the intricacies between green growth and its dynamics, a handful of studies have been dedicated to understanding both economic and noneconomic factors influencing green growth. In an empirical articulation, Le et al.^[^ ^36^ ^]^ submitted that good governance is a precursor to green growth with significantly varying effects across high, middle, and low‐income countries. This submission highlights country‐specific nuances that must not be neglected for streamlined policy administration within a specific geography. Hence, the choice of the US economy is considered critical given its vintage position among other global economies. In the cases of Nepal and Bangladesh, Baniya et al.^[^ ^23^ ^]^ emphasized the deleterious and supportive effects of structural changes and renewable energy consumption on green growth, respectively. The perspectives of information and communication technology (ICT), economic policy uncertainty (EPU), and financial risks were extended by Ofori et al.,^[^ ^37^ ^]^ Sohail et al.,^[^ ^14^ ^]^ and Zhao et al.^[^ ^38^ ^]^ These studies inferred the improving effects of ICT, whereas EPU and financial risks are detrimental to green growth in sub‐Saharan Africa, as well as high‐polluting countries. Irrespective of their specific or multiple country inclinations, these studies failed to consider the likely implications of energy vulnerability and energy uncertainty on green growth. Meanwhile, the perspectives of recent studies are summarized in **Table** **1**.

**Table 1:** Perspectives of recent studies on green growth predictors.

Author(s)	Scope	Model	Predicting variables	Effects
Sun et al.^[^ ^39^ ^]^	47 countries (1996–2018)	FE, SYS‐GMM, PQREG	Financial risk, GDP, Trade openness, capital formation, population	Financial risk, trade openness, and population (−) GDP and capital formation (+)
Borojo et al.^[^ ^24^ ^]^	25 emerging economies (1991–2019)	PMG, AMG‐CSADL	EPU, energy consumption, green innovation, and governance.	EPU and energy consumption (−) Innovation and good governance (+)
Cheng et al.^[^ ^40^ ^]^	Central Asia (1995–2021)	ARDL	Mineral resources, hydropower, and urbanization	Mineral resources and urbanization (−) Hydropower (+)
Dong et al.^[^ ^41^ ^]^	30 Chinese provinces (2014–2018)	RE, FGLS, Dif‐GMM, Sys‐GMM	Low‐carbon energy, energy efficiency	+
Dong & Ullah^[^ ^42^ ^]^	China (1997–2021)	ARDL, QARDL	Internet of Things, environmental regulations, renewable energy, and R&D.	+
Huang^[^ ^43^ ^]^	China (2000–2017)	FE, IV‐GMM, IV‐Tobit	Trade, fiscal decentralization, and renewable energy.	+
Jia et al.^[^ ^44^ ^]^	30 Chinese provinces 10 years	SDM, DSDM	Independent innovation, imitation innovation	Independent innovation (+) Imitation innovation (−)
Jiang et al.^[^ ^45^ ^]^	E7 countries (1996–2019)	PQR	EPU, institutional quality, and renewable energy.	EPU (−) Institutional quality and renewable energy (+)
Jin et al.^[^ ^46^ ^]^	15 APEC countries (2010–2021)	FMOLS	Energy resources and green finance	Energy resources (−) Green finance (+)
Liu et al.^[^ ^5^ ^]^	Top emitter countries (1991–2020).	CS‐NARDL	Fiscal policy shocks and financial development	Positive fiscal policy shocks (+) Negative fiscal policy shocks (#) Financial development (+)
Rasheed et al.^[^ ^47^ ^]^	IEA countries (1990–2020)	PMG, DOLS, FMOLS	EPU and EPS	EPU (−) EPS (+)
Razzaq, Afshan, et al.^[^ ^48^ ^]^	37 IAE countries (2007–2020)	DPTM	Energy transition, environmental governance.	Energy transition is negative in some countries and positive in others. Environmental governance (+)
Razzaq, Skare, et al.^[^ ^49^ ^]^	Chinese regions (2007–2019)	MMQR, GMM	Digital finance and renewable energy technology	Varying positive and negative effects across the quantiles.
Ren et al.^[^ ^50^ ^]^	20 polluted countries (2007–2020)	OLS, 2‐SLS, GMM	Supply chain and ICT	+
Shang et al.^[^ ^51^ ^]^	Asian economies (2000–2021)	PMG	Tourism, energy resources	Tourism benefits green growth in high‐income and impedes the same in low‐income Asian countries. Energy resources (−)
Tang et al.^[^ ^52^ ^]^	BRICS (1993–2020)	ARDL	ICT, financial development	ICT improves green growth in all BRICS countries except India. Financial development (+)
Wang, Huang, et al.^[^ ^53^ ^]^	Knowledge‐based economies (1990–2019)	CS‐ARDL	ICT, natural resources, capital formation, labor force, exports, fossil fuels	ICT, natural resource, capital formation, and labor force (+) Exports (−)
Wang, Peng, et al.^[^ ^54^ ^]^	BRICS (1990–2019)	MMQR	Renewable energy, ecological governance, human development, R&D expenditure	+
Xiaofang et al.^[^ ^55^ ^]^	China (1990–2020)	WQC	EPU, GPR	–
Xu & Li^[^ ^56^ ^]^	30 Asian economies (2010–2021)	FMOLS	Resource efficiency, governance, fossil fuel, green finance	Resource efficiency, governance, and green finance (+) Fossil fuels (‐)
Yao^[^ ^57^ ^]^	BRICS (1990–2020)	CS‐ARDL	Tourism, capital formation, FDI, human capital	Tourism and human capital (+) FDI and capital formation (−)
Yuan et al.^[^ ^58^ ^]^	China (2010–2021)	DEA	Energy efficiency, tax, and subsidies	+
Zhang et al.^[^ ^59^ ^]^	Selected Asian economies (1995–2020)	QARDL	ICT, financial fragility, education	ICT and education (+) Financial fragility (−)
Zheng et al.^[^ ^13^ ^]^	China (1996–2020)	ARDL, QARDL	ICT, financial integration	+
Abbas et al.^[^ ^25^ ^]^	12 developing countries (2004–2023)	FMOLS	Financial inclusion, green innovation, FDI, trade openness	Financial inclusion (−) Green innovation, FDI, and trade openness (+)
Agan & Balcilar^[^ ^60^ ^]^	38 OECD countries (1990–2020)	GMM	Green technology, climate adaptation, governance	+
Anwar et al.^[^ ^26^ ^]^	Selected fragile nations (1996–2019)	PQR, DOLS, FMOLS	EPU, financial development, green innovation	+
Bakhsh et al.^[^ ^61^ ^]^	Mineral‐endowed countries (2000–2021)	MMQR	Digital finance, resource consumption, technology, human capital	Digital finance (+) Resource consumption (−) Technology and human capital (asymmetry)
Caijuan et al.^[^ ^29^ ^]^	G7 countries (1990–2020)	PQR	GPR, EPS	GPR (−) EPS (+)
Citil^[^ ^27^ ^]^	G20 countries (2004–2020)	PQR	Green finance, environmental taxes, ecological regulations	+
Feng et al.^[^ ^62^ ^]^	BRICS (1995–2021)	PNARDL	Resource volatility, green innovation, open trade, EPS	Resource volatility (−) Green innovation, trade openness, and EPS (+)
Guo et al.^[^ ^63^ ^]^	17 EAP countries (2000–2020)	CS‐ARDL	Resource efficiency, technological maturity, green finance	Resource efficiency and green finance (+) Technological maturity (−)
Hu & Gu^[^ ^64^ ^]^	10 copper producing countries (2000–2020)	GMM	Resource scarcity, FDI, financial dynamics, tax revenue	Resource scarcity and tax revenue (−) FDI and financial dynamics (+)
Li et al.^[^ ^30^ ^]^	China (1990–2021)	WQC	Mineral rents, GPR, EPU	GPR and EPU (−) Mineral rents (#)
Liu & Chen^[^ ^65^ ^]^	Asian knowledge‐based countries (1990–2019)	AMG, CCEMG	Natural resources and green economy	+
Si et al.^[^ ^66^ ^]^	China (2003–2018).	Fixed effects	Green technology, natural resources	Green technology (+) Natural resources (−)
Sun et al.^[^ ^39^ ^]^	China urban agglomeration (2013–2020)	IV‐2SLS	Digital finance, human capital	+
Tan et al.^[^ ^67^ ^]^	China (1996–2020)	ARDL, QARDL	Tourism, ICT, financial development	+
Tariq et al.^[^ ^68^ ^]^	12 Emerging economies (1996–2021)	CS‐NARDL, NAMG,	Eco‐innovation, green trade openness, carbon price, green energy	+
Tufail et al.^[^ ^69^ ^]^	19 OECD countries (1991–2021)	MMQR	Green finance, human capital, globalization, GDP	Green finance and human capital (+) Globalization and GDP (−)
Wei^[^ ^70^ ^]^	17 high‐income and 15 low‐income countries (2005–2021)	SYS‐GMM	Mineral resources trade, urbanization, electricity consumption, poverty, internet use	Mineral resource and internet use (+) Urbanization, electricity, and poverty (−)
Xiaoping & Yanqiu^[^ ^71^ ^]^	11 East Asian economies (1998–2021)	PMG, CS‐ARDL	Fuel efficiency, internet access, GDP, FDI	Fuel efficiency, internet access, and GDP (+) FDI (−)
Yang et al.^[^ ^72^ ^]^	South Asia countries (1990–2021)	CS‐ARDL, AMG	Digitalization, human and financial development, institutional efficiency, mineral resources	Mineral resources (−) Digitalization, human and financial development, and efficient institution (+)
Yu et al.^[^ ^73^ ^]^	China (1980–2021)	Entropy‐weighted TOPSIS	Resource rent, economic risk, governance, IT expenditure	Economic risk, IT expenditure (−) Resource rent and governance (+)

AMG, PMG, ARDL, FE, RE, DOLS, FMOLS, NARDL, SYS‐GMM, CS‐ARDL, MMQR, CS‐NARDL, NAMG, QARDL, IV‐2SLS, WQC, PQR imply augmented mean group, pool mean group, autoregressive distributed lag, fixed effects, random effects, dynamic ordinary least squares, fully modified ordinary least squares, nonlinear ARDL, system generalized method of moments, cross‐sectional ARDL, method of moment quantile regression, cross‐section NARDL, nonlinear AMG, quantile ARDL, instrumental variable 2‐stage least squares, wavelet quantile correlation, and panel quantile regression.

+, −, and # connote positive, negative, and heterogeneous effects, respectively.

The holistic review of extant literature revealed some humongous gaps in the literature requiring urgent empirical verification to support green growth initiatives in the US and other emerging economies. A notable drawback of prior studies is that the peculiarity of a country like the US was not adequately evaluated despite its position as a global leader in green initiatives. Most available studies addressed the peculiarities of China and other Asian nations. Another critical gap in extant studies is the absence of any study verifying the effects of energy vulnerability on green growth in any economy. Such negligence on a factor with strong potential effects on environmental quality has strong policy‐limiting implications. There is a need to uncover the specific implications of energy vulnerability on green growth, particularly in the US, given its strong energy vulnerability resiliency. Besides, these studies extended countervailing reports about the implications of the respective factors on green growth in several economies. Such conflicting submissions also undermine policy administration. Further insights highlight that most prior studies relied on pre‐COVID‐19 and pre‐Russian‐Ukraine conflict data sets. The outcome of such empirical exercise may not apply to the current economic realities, undermining policy moderation for green growth both for the US and other economies. Not least, the time‐quantile‐frequency response of green growth to the selected predictors is conspicuously absent in empirical literature since they relied on techniques lacking the flexibility to unravel such peculiarities. Having noted the limitations of existing studies, the current investigation harnessed relevant variables and robust estimators for updated policy insights that could ensure green growth in the US.

## 3 Experimental Section

This component of the study consists of three sub‐sections, including the description of data and sources, model specification, and estimation techniques.

### 3.1 Data Description and Sources

The empirical analysis of this study relied on monthly time series data (2000m1–2022m12) extracted from reliable data repositories. The choice of this period is a function of data availability and consistency. The dependent variable (green growth), energy vulnerability, digitalization, and energy efficiency are originally annual series but converted to monthly through the quadratic‐match‐sum procedure. The quadratic‐match‐sum procedure ensures the smooth and plausible conversion of low‐frequency series to high‐frequency series by dropping end‐to‐end data variabilities.^[^ ^74^, ^75^ ^]^ More details about the variables are elicited in **Table** **2**.

**Table 2:** Data descriptions.

Series	Notation	Measurements	Sources
Green growth	GRG	Global Green Growth Index	https://greengrowthindex.gggi.org/
Energy vulnerability	ENV	Energy security index	IEA
Energy uncertainty	EUC	Energy‐related uncertainty index	Dang et al. (2023)
Energy Efficiency	EEF	GDP/Energy Use	Authors
Digitalization	Digit	ICT adoption	OWID
Geopolitical risk	GPR	GPR	https://www.matteoiacoviello.com/gpr.htm

IEA and OWID denote the International Energy Agency and Our World in Data, respectively.

### 3.2 Model Specification

The study's specific objective is to evaluate the implications of energy vulnerability and the other enlisted series on green growth in the US. Hence, green growth (GRG) represents the response variable. Conversely, energy vulnerability is the major explanatory variable, while energy uncertainty and geopolitical risk factors are the supporting explanatory variables. We enlisted energy efficiency and digitalization as control variables based on insights gained from existing literature.^[^ ^71^, ^76^ ^]^ These series strongly affect environmental performance and sustainability.^[^ ^72^, ^77^, ^78^ ^]^

Based on insights drawn from Sohail et al.^[^ ^14^ ^]^ and Anwar et al,^[^ ^26^ ^]^ the classical production function is hereby specified to study the relationship between green growth and the listed predictors. The traditional production function is illustrated in Equation (1).

(1) $$Y_{t}=\left(L_{t},K_{t}\right)$$

*Y_t_* in Equation (1) represents economic growth that may be sustainable (green) or otherwise. *L_t_*, *K_t_* Denote labor and capital, respectively. This study explored how the earlier highlighted factors can predict sustainable growth (green growth). The implicit equation to predict this dynamic relationship is provided in Equation (2). The conjecture is premised on the reported implications of these series on environmental performance.^[^ ^72^, ^77^, ^78^ ^]^

(2) $$GRG_{t}=f\left(ENV_{t},EUC_{t},GPR_{t},EEF_{t},DGT_{t}\right)$$

In Equation (2), *GRG_t_* depicts green growth (dependent variable), *ENV_t_*, *EUC_t_*, *GPR_t_*, *EEF_t_*, *DGT_t_* energy vulnerability, energy uncertainty, geopolitical risk, energy efficiency, and digitalization, respectively. *f* is the functional notation illustrating the predicted relationship. In Equation (3), an explicit econometrics equation illustrates the predicted marginal effects of the listed explanatory variables on green growth.

(3) $$\begin{array}{ccc} GRG_{t} & = & \rho_{0}+\rho_{1}ENV_{t}+\rho_{2}EUC_{t}+\rho_{3}GPR_{t}+\rho_{4}EEF_{t}\\ & & +\;\rho_{5}DGT_{t}+\varepsilon_{t} \end{array}$$

In Equation (3), ρ_0_ denotes the intercept, ε_*t*_ depicts the stochastic error component, while the superscript *t* denotes the time frame. Likewise, the coefficients of the respective explanatory variables are given by ρ_1_, ρ_2_, ρ_3_, ρ_4_, ρ_5_, respectively. Among the enlisted series, energy vulnerability, energy uncertainty, and geopolitical risks are presumably green growth inhibitors. Conversely, energy efficiency and digitalization are probable green growth enablers. These probable relationships are illustrated mathematically below:

(4) $$\begin{array}{ccc} \rho_{1} & = & \frac{\Delta GRG_{t}}{\Delta ENV_{t}}<0,\;\rho_{2}=\frac{\Delta GRG_{t}}{\Delta EUC_{t}}<0,\;\rho_{3}=\frac{\Delta GRG_{t}}{\Delta GPR_{t}}<0,\\ \rho_{4} & = & \frac{\Delta GRG_{t}}{\Delta EEF_{t}}>0,\;\rho_{5}=\frac{\Delta GRG_{t}}{\Delta DGT_{t}}>0 \end{array}$$

### 3.3 Estimation Procedures

The novel empirical estimates of the current engagement emerged by implementing three novel time‐varying econometrics protocols. The motivation is to produce updated empirical analysis that will improve the green growth prospects of the US and likely other countries desirous of improved environments. Herein, the three novel techniques include the Wavelet Quantile Regression (WQR), the Wavelet Quantile Granger Causality (WQGC), and the Rolling Windows Wavelet Quantile Granger Causality (RWWQGC).

### 3.4 Wavelet Quantile Regression (WQR)

The recently introduced WQR is a notable and robust econometric technique that considers time and quantile effects among variables. This novel technique, attributable to Kumar & Parakandla,^[^ ^79^ ^]^ follows the traditional quantile regression procedure of Percival & Walden.^[^ ^80^ ^]^ Dissimilar to traditional econometric tolls, the WQC technique accounts for potential asymmetry, time‐varying, and nonlinear implications of explanatory series over the quantile distributions of the response series.^[^ ^81^, ^82^ ^]^ The technique ensures accuracy and precise empirical articulations in the face of outliers. Such attributes stem from its capacity to consider tail and structured interactions over different horizons.^[^ ^81^, ^82^ ^]^ The mathematical scheme of the novel WQC technique is illustrated in Equation (5) accordingly.

(5) $$WQC_{\pi }\left(d_{j}\left[X\right],d_{j}\left[X\right]\right)=\frac{q{cov}_{t}\left(d_{j}\left[X\right],d_{j}\left[X\right]\right)}{\sqrt{var\left(\theta_{t}\left(d_{j}\left[Y\right]-Q_{\pi ,d_{t[Y]}}\right)\right)var\left(d_{j}\left[X\right]\right)}}$$

where *X* is the selected predicting variable, and *Y* is the response variable (green growth). Following this protocol, the quantile implications of energy vulnerability and other enlisted variables on green growth would be elicited for the immediate, medium, and longer time dimensions.

### 3.5 Wavelet Quantile Granger Causality (WQGC)

Closely related to the *WQC* procedure, the novel *WQGC* estimator proposed by Ozkan et al.^[^ ^9^ ^]^ explores the causal effect relationship among series over different horizons. Accordingly, the technique examines causal interactions between two variables across time and quantile dimensionalities. The WQGC is superior to existing causal evaluation techniques that observe such effects in isolations. Considering the superiority of this technique, the causal effects of energy vulnerability and other selected variables on green growth in the US would be ascertained to make room for streamlined policy administration since prior studies are devoid of such information. Meanwhile, more insights about the methodological protocols of the WQGC can be obtained from the original study of Ozkan et al.^[^ ^9^ ^]^

(6) $$d_{z}{\left[Y\right]}_{t}^{\pi }=\partial_{z,\pi }+\sum_{u=1}^{m}\delta_{z,\pi ,\mu }d_{z}{\left[Y\right]}_{t-\mu }^{\pi }+\sum_{u=1}^{m}\beta_{z,\pi ,\mu }d_{z}{\left[X\right]}_{t-\mu }^{\pi }+\varepsilon_{t,z,\pi }$$

where ∂_*z*,π_ indicates the constant parameter at the π‐*th* conditional quantile, and at *z*‐*th* the decomposition point. $d_{z}{\left[Y\right]}_{t}^{\pi }$ represents the π‐*th* conditional quantile of the *z*‐*th* decomposition of the dependent variable. *d_z_* [*X*] demonstrates the Granger causal potential of the predicting variable at the *z*‐*th* decomposition. Following these protocols, the null hypothesis is rejected at the π‐*th* conditional quantile which β_*z*,π,µ_ is significantly different from zero. At such *z*‐*th* decomposition, the explanatory variable is said to Granger cause the response variable.

### 3.6 Rolling Windows Wavelet Quantile Granger Causality (RWWQGC)

The empirical estimates of this study evolved through other robustness tests through the application of the recently introduced RWWQGC. The newly proposed technique (Ref. [83]) complements the outcomes of both the WQC and the WQGC protocols by producing the time‐quantile‐frequency interactions of the relevant series. Its ability to unravel the dynamic interactions across time, frequencies, and quantiles separates its outcome from the isolated approaches of others. Enthusiastic readers could gain more insights into this unique econometric technique from Adebayo et al.^[^ ^84^ ^]^

(7) $$\begin{array}{ccc} RWWQGC(X\to Y) & = & a_{w,x,\pi }+\sum_{b=1}^{B}\delta_{b}d_{x}{\left[Y_{1}^{\pi }\right]}_{t-b}+\sum_{b=1}^{B}\gamma_{b}d_{x}{\left[X^{\pi }\right]}_{t-b}\\ & & +\;u_{w,x,\pi } \end{array}$$

**Figure 2:**
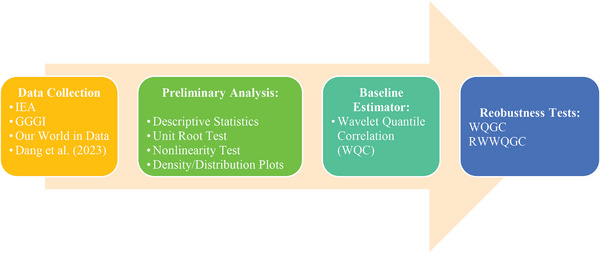
Methodological flow.

In Equation (7), *w*, *x*, π denote the overlapping rolling windows, the decomposition positions, and quantiles, respectively. Likewise, *a*, *b*, *u* represent constant, lag lengths, and the standard error, respectively. The methodological flow of the empirical procedures is illustrated in **Figure** **2**.

## 4 Data Analysis and Discussion

**Figure 3:**
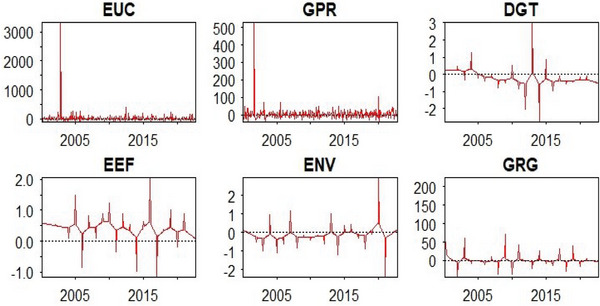
Distributions of series.

Relevant preliminary tests, including summary statistics, distribution plots, stationarity, and nonlinearity tests, precede the empirical analysis provided in this study. The outcomes of the summary statistics are provided in **Table** **3**. This is followed by the distribution plots of all the enlisted series in **Figure** **3**.

**Table 3:** Summary statistics.

	GRG	ENV	EUC	GPR	EEF	DGT
N	273	273	273	273	273	273
Mean	0.0345	105	16.4	0.502	2.05e+7	1.43
Median	0.0323	111	15.1	0.444	1.52e+7	1.52
Standard deviation	0.00784	17.3	9.67	0.246	1.12e+7	0.623
Minimum	0.0206	77.8	0.00	0.138	7.61e+6	0.237
Maximum	0.0504	127	57.9	1.83	4.35e+7	2.34
Skewness	0.202	−0.409	0.939	1.65	0.573	−0.345
Std. error skewness	0.147	0.147	0.147	0.147	0.147	0.147
Kurtosis	−1.13	−1.43	1.51	4.04	−1.23	−0.966
Std. error kurtosis	0.294	0.294	0.294	0.294	0.294	0.294
Shapiro‐Wilk W	0.954	0.861	0.952	0.876	0.852	0.944
Shapiro‐Wilk p	< .001	< .001	< .001	< .001	< .001	< .001

The evidence emanating from Table 3 underscores that energy vulnerability (ENV) and energy uncertainties (EUC) are more dispersed than others. Conversely, green growth (GRG) is the least dispersed variable. Furthermore, the summary statistics indicate that all the series, except EUC and GPR, have negative tails. More insights based on the Shapiro‐Wilk probability values underscore that all the series are non‐normally distributed. This inference is also supported by the variables distribution plots (Figure 3). Hence, further steps, including linearity and stationarity tests, were explored. Accordingly, **Tables** **4** and **5** summarize the outcomes of the nonlinearity and stationarity tests, respectively. The study selected the BDS procedure to verify nonlinearity issues, whereas the traditional ADF and nonlinear procedure of Lee‐Strazicich LM were selected to verify unit‐root problems. The outcomes of these preliminary tests validate the choice of time‐sensitive estimators. Meanwhile, the application of robust estimators that produce such empirical details across frequencies and time‐quantile horizons is particularly noteworthy, given the non‐existence of such narratives in extant studies.

**Table 4:** BDS nonlinearity test.

Dimension	GRG	ENV	EUC	GPR	EEF	DGT
2	0.205^a)^	0.201^a)^	0.202^a)^	0.178^a)^	0.204^a)^	0.198^a)^
3	0.346^a)^	0.337^a)^	0.336^a)^	0.294^a)^	0.346^a)^	0.333^a)^
4	0.444^a)^	0.430^a)^	0.429^a)^	0.368^a)^	0.443^a)^	0.424^a)^
5	0.511^a)^	0.494^a)^	0.494^a)^	0.412^a)^	0.509^a)^	0.487^a)^
6	0.558^a)^	0.538^a)^	0.541^a)^	0.440^a)^	0.553^a)^	0.530^a)^

^a)^ Implies the rejection of the null hypothesis of linearity at a 1% significance level.

**Table 5:** Stationarity test.

Series	ADF		Lee Strazicich LM unit root test			
	Level	1st difference	Level	Breakpoint	1st difference	Breakpoint
GRG	4.659^a)^	–	−4.003^a)^	2002M12	–	–
ENV	−2.503	−5.248^a)^	−1.712	2003M01	−5.595^a)^	2011M12
EUC	−9.406^a)^	–	−7.326^a)^	2012M06	–	–
GPR	−6.088^a)^	–	−3.186	2019M12	−13.320^a)^	2008M06
EEF	0.939	−5.313^a)^	−2.236	2015M12	−5.305^a)^	2007M12
DGT	1.080	−4.393^a)^	−1.026	2008M01	−6.177^a)^	2004M11

^a)^ Implies the rejection of the null hypothesis of non‐stationarity at a 1% significance level.

### 4.1 Estimates of the Wavelet Quantile Regression

**Figure 4:**
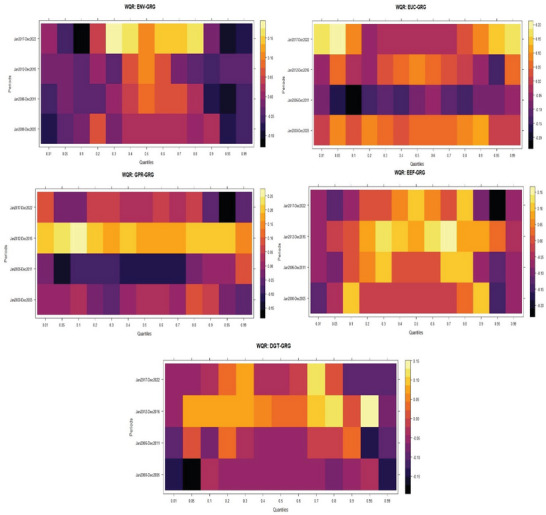
Wavelet quantile regression plots.

The wavelet quantile regression heatmap (**Figure** **4**) divulges some notable issues worth highlighting at the outset. First, the heatmaps illustrate the connectedness of green growth and its selected determinants over four time dimensions, including the immediate, medium, and longer timers. Through this, the study can predict the actual effects of each explanatory factor on the response factor. Such inferences would help policymakers understand the relationship's peculiarity at each epoch and craft applicable policy options to address such peculiarities. Furthermore, the heatmap's extreme yellow boxes represent positive interactions between the predicting and predicted variables, whereas the extreme black boxes represent negative interactions.^[^ ^81^, ^82^ ^]^

On the relationship between energy vulnerability and green growth, the heatmap (WQC: GRG‐ENV) underscores notable heterogeneous and asymmetric connectedness across the quantile distributions of green growth. At the immediate time (January 2000–December 2005), energy vulnerability aggravated environmental degradation by reducing green growth at the lower and upper quantiles of the distributions. However, its effects were neutral at the middle quantiles of green growth distributions. Energy vulnerability replicated similar outcomes at the lower‐medium horizon (January 2006–December 2011) of the distributions. The environmental reducing effects of energy vulnerability became more pronounced at the upper‐medium horizon (January 2012–December 2016), where it most significantly reduced green growth, particularly in the lower and upper‐quantiles of the distributions. Interestingly, there are notable twists of events at the upper horizon (January 2017–December 2022). Energy vulnerability within this horizon (longer term) produced substantial green growth‐enhancing effects across the distributions. Notably, green growth initiatives produced profound energy vulnerability resistance effects between the lower‐middle and lower upper‐quantiles (q0.30‐q80) of the distributions in the long term.

The observed relationship between green growth and energy vulnerability underscores strong policy implications for environmental sustainability in the US. In tandem with the submissions of Borozan^[^ ^85^ ^]^ and Ozkan et al.,^[^ ^9^ ^]^ policy administrators must pay adequate attention to changing economic events to effectively address the varying effects of energy vulnerability on green growth. The observed heterogeneous and asymmetric interactions emanate from the changing economic occurrences directly impacting the environment. Some proactive policy measures are essential to curtail energy vulnerability's short‐ and medium‐term profound negative effects. Ensuring an adequate supply of energy at all times and reducing reliance on energy importation are essential steps to mitigating the pressures of energy vulnerabilities. As reflected in Figure 2, the US's energy self‐sufficiency and least dependence on external sources paid off in the long term with notable positive effects on green growth. This outcome contradicts the submissions of Ozkan et al.^[^ ^9^ ^]^ in the case of Turkey, where energy vulnerability reduces the load capacity factor (LCF) more profoundly in the longer term. The outcome rather reflects the US's effective and consistent implementation of policies that ensure reliance on externally sourced energy. Hence, it is recommended that more policy actions be extended to reduce the immediate and medium‐term negative effects of energy vulnerability on environmental sustainability.

Likewise, the heatmap (WQC: GRG‐EUC) unveils the relationship between energy uncertainty and green growth with notable policy insights for environmental sustainability in the US. Like the effects of energy vulnerability, energy uncertainties produced notable asymmetric effects across the quantile distributions of green growth. Their effects vary across the quantiles of green growth in short, medium, and longer terms. The observed heterogeneous effects highlight that green growth is achievable irrespective of energy uncertainties with policy consistencies that reduce the negative effects. Specifically, the negative effects of energy uncertainties were more profound at the 0.10 quantile of the distributions of green growth at the lower medium term. Conversely, green growth progresses during the upper medium and long term of the distributions amidst energy uncertainties. Among all, the most appealing effects were recorded at the 0.01 and 0.05 quantiles in the long term of the distributions of green growth. This is followed by some improving effects at the 0.95 and 0.99 quantiles of the distributions during the longer and upper‐medium terms.

Comparatively, green growth initiatives show more resilience to the impasse of energy uncertainties than energy vulnerabilities. Hence, ensuring energy self‐sufficiency and mainstreaming critical stabilizers and other essential buffers that pertain to the energy sector would keep the US environment clean by reducing the negative repercussions of energy uncertainties. Given that extant studies like Dang et al.,^[^ ^4^ ^]^ emphasized that energy uncertainties tend to limit sustainable development, it is expedient to forestall its effects at all times. As earlier highlighted, policymakers must pay attention to the short‐term profound negative effects by providing necessary short‐term buffers. A possible policy option is to deepen the depth and spread of renewable energies. The availability and affordability of alternative energies would be able to protect the economy from the immediate shocks arising from energy uncertainties.

The third heatmap (WQC: GRG‐GPR) illustrates the implications of GPR on green growth. The heatmap highlights strong evidence of prominent asymmetric affiliations between GPR and green growth across the entire distribution. The heatmap indicates that GPR hampered environmental sustainability by lowering green growth in the immediate and longer‐term across the quantiles of the distributions. Specifically, the negative effects became more pronounced at the lower quantile (*q5‐q70*) and relatively strong at the upper quantile (*q95*) of the distributions of green growth. Beyond this point, green growth emerged stronger with some substantial resistance to GRG across all quantile distributions during the upper medium terms. During this period, policy initiatives were able to forestall the observed short‐term negative effects of GPR. Herein, green growth showed more resistance to GPR between January 2012 and December 2016. This period could be regarded as a recovery period after the global financial crises of 2006–2008. Unfortunately, the recovery and resiliency to the vagaries of GPR were not sustained till 2017–2022. Environmental quality suffered substantial reductions during this time due to rising geopolitical tensions. Among such crises that warranted the heightened GPR and consequential environmental degradations are the COVID‐19 global pandemic and the Russian invasion of Ukraine. These global events translate to reduced green growth in the US, particularly at the 90th, 95th, and 99th quantiles of the distributions of green growth.

In conformity with the submissions of prior investigations in the cases of G7 (Caijuan et al.^[^ ^29^ ^]^) and China (Li et al.^[^ ^30^ ^]^), GRP resistance policy buffers are critical to curtailing the green growth disruptive effects of GPR at all times. Likewise, in tandem with the reports of Xiaofang et al.^[^ ^55^ ^]^ in the case of China, the resistance and resilience of green growth to the vagaries of geopolitical shocks should be extended for all‐time environmental sustainability.

The fourth heatmap (WQC: GRG‐EEF) depicts the interactions of energy efficiencies and green growth across the distributions during the short, medium, and longer horizons. Like the implications of other enlisted series, energy efficiency produced heterogeneous and prominent asymmetric effects on green growth. However, it is worth noting that the available evidence portrays that energy efficiency produced more green growth‐enhancing effects than other predictors of green growth in the US. The green growth‐enhancing effects of energy efficiency are recorded across the four selected horizons with isolated unpleasant effects. Specifically, energy efficiency dampened green growth more profoundly at the 0.95 quantile in the longer horizon. Its profound negative effects were also recorded at the 0.01 and 0.95 quantiles in the immediate time, the 0.05 and 0.95 at the lower‐middle horizon, and the 10th quantile in the long term. The policy thrust of the observed relationship between energy efficiency and green growth in the USA underscores the importance of efficient utilization for environmental prosperity. The ability of energy efficiency to evolve green growth and environmental vitality has been articulated in some extant investigations, including Dong et al.^[^ ^41^ ^]^ and Yuan et al.^[^ ^58^ ^]^ for China, and Uche, Okere & Das^[^ ^82^ ^]^ in the case of India. Hence, it behooves the US environmental administrators to push the depth of energy efficiency to attain relevant, sustainable goals and to make the environment friendlier. Certainly, efficient energy utilization and the adoption of more energy‐saving devices remain critical for environmental sustainability.

Not least, the fifth heatmap (WQC: GRG‐DGT) illustrates the dynamic interactions of digitalization and green growth across the distributions during the short, medium, and longer dimensions. Heterogeneous and asymmetric effects characterized the relationship between digitalization and green growth. These outcomes require specific policy options to address the peculiarities of changing economic realities at each stage of the distribution. The evidence portrayed in the heatmap underscores green growth‐reducing effects, especially in the short term, and more green growth‐enhancing effects in the long term. Between January 2000 and December 2005 (immediate term), digitalization dampened green growth more profoundly at the 0.01, 0.05, and 0.99 quantiles of green growth distributions. Similar green growth disruptive effects were recorded at the 0.01, 0.10, 0.95^,^ and 0.99 quantiles from January 2006 to December 2011. This could be likened to incubation periods during which digitalization poses more harm to the environment due to its capital‐intensive nature. During such time, policymakers struggle to improve the depth and penetration of digitalization by harnessing available resources, leaving untold pressure on the environment. Hence, short‐term green growth‐dampening effects of digitalization might be inevitable. However, with proactive planning, policymakers can effectively reduce its negative effects.

Interestingly, there is a turn in events during the upper‐medium (January 2012–December 2017) and long‐run (January 2017–December 2022). Within this horizon, digitalization produced substantial green growth‐enhancing effects. Although there are instances where digitalization constrained green growth (0.01 and 0.99) in the upper‐medium and 0.01, 0.05th, 0.80‐0.95 quantiles in the longer term, its effects are predominantly appealing to green growth. Inferences from related extant investigations^[^ ^41^, ^66^, ^73^ ^]^ underscore such varying effects where digitalization improved environmental quality only in the long term. Likewise, Tang et al.^[^ ^52^ ^]^ emphasized that some aspects of the digitalization process remained inimical to green growth in the BRICS economy, even in the long term. Given such attributes, policymakers must be circumspect when deploying digitalization infrastructure. More emphasis should be placed on environmentally sensitive digital infrastructures for improved green growth.

### 4.2 The Wavelet Quantile Granger Causality Analysis

**Figure 5:**
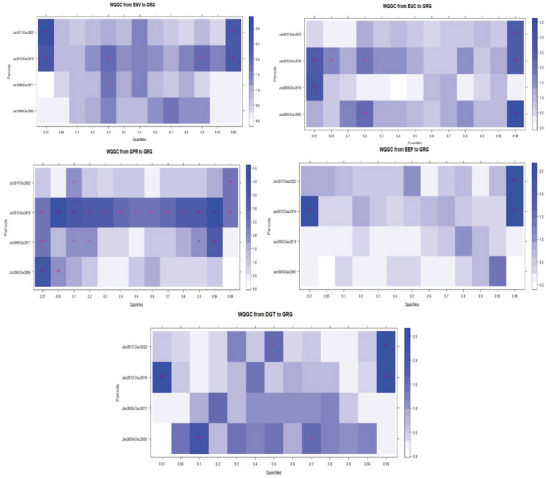
Wavelet quantile Granger causality plots. Note: *, and ** denote the rejection of the null hypothesis at 5% and 1% significance levels.

**Figure 6:**
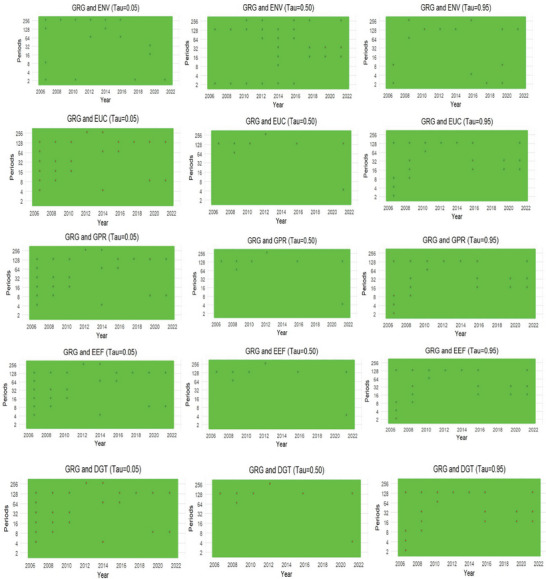
Rolling windows wavelet quantile Granger causality plots. Note: * implies the rejection of no Granger causal effects at the 5% significance level. Windows size = 250.

The estimates of the wavelet quantile regression were subjected to some robustness tests with novel wavelet quantile Granger causality (WQGC) and the novel rolling windows wavelet quantile Granger causality (RWWQGC) techniques. Herein, **Figure** **5** depicts the heatmaps illustrating the causal relationships between green growth and the enlisted determinants within a wavelet protocol. Furthermore, **Figure** **6** contains the heatmaps illustrating the causal relationships between green growth and the enlisted determinants within the rolling windows wavelet procedures. As stated earlier, while the WQGC identifies the dynamic causal relationships across time and quantiles, the RWWQGC accounts for dynamic causal relationships across time, quantiles, and frequencies. The application of these novel Granger causality techniques enhanced the understanding of the causal effects between the selected predictors and green growth in the US during the studied period.

### 4.3 The Rolling Windows Wavelet Quantile Granger Causality Analysis

The outcomes of the novel wavelet quantile Granger causality and the novel rolling window wavelet quantile Granger causality largely aligned with the evidence portrayed by the wavelet quantile regression. Particularly, these novel nonparametric Granger causality protocols underscore the heterogeneous and asymmetric implications of the enlisted variables on green growth in the US. The outcomes also highlight the causal effects in different times, quantiles, and frequencies. These discoveries provide critical empirical evidence for US policy administrators to counteract the implications of energy variabilities and geopolitical risks on the local economy. Likewise, the evidence underscores the essence of a robust energy efficiency path and digitalization infrastructure for sustained green development in the US. Yet, the evidence illustrated in the nonparametric Granger causality procedures also reinforced the earlier submission, cautioning the formulation and implementation of a one‐size‐fits‐all policy stance. In this regard, energy vulnerability Granger caused green growth most profoundly at the two extreme quantiles (0.01q and 0.099q) during the upper‐medium term (January 2012–December 2016) and the longer term (January 2017–December 2022). On the other hand, energy uncertainty Granger caused green growth mainly at the extreme quantiles across the time distributions of green growth. Geopolitical risk factors Granger caused green growth most profoundly during the immediate and upper‐medium terms, with the least Granger causal effects in the long term. This particular outcome also reinforces the initial discovery of the implications of geopolitical risks on green growth in the US.

The Granger causal effects of energy efficiency and digitalization infrastructures are not different from those of other enlisted series. Particularly, energy efficiency Granger caused green growth most profoundly at the extreme quantiles (0.01q and 0.099q) during the upper‐medium and longer terms. Conversely, digitalization Granger caused green growth over the entire time dimension. However, its Granger causal effects were more profound at the extreme quantiles during January 2012–December 2016 and January 2017–December 2022. The imports of these empirical discoveries entail that these enlisted variables are critical determinants of green growth in the US. Hence, policymakers must pay adequate attention to ensuring the realization of the country's green growth initiatives and the United Nations’ sustainable development goals 7 and 13. Besides, the outcomes call on policymakers to craft specific policy options to address the peculiarities of each factor, given their varying effects on green growth during the studied period.

The illustrated corroborating evidence is further highlighted by the outcomes of the RWWQGC heatmaps (Figure 6). The heatmaps reveal that Granger causal effects of energy vulnerability are strongest during the longer time frequencies with more profound effects at the 0.50 quantiles from 2014 to 2022. The Granger causal effects of other variables are similar, with the most profound implications at the extreme quantiles (0.01q and 0.95q) over the entire frequency and quantile distributions of green growth.

## 5 Conclusion and Policy Recommendations

This study is conceived to fill some yarning gaps in the literature pertaining to the determinants of green growth in the US. Prior studies ignored the perspective of energy vulnerability as a potential determinant of green growth in any country. Likewise, they ignored the perspective of the US, with more attention paid to the Chinese economy. The US is, unarguably, the leading global economy and the second‐largest energy consumer. Hence, ignoring the peculiarities of such an economy would conceal and constrain empirical insights to ensure sustainable development for the US and other economies. Hence, this study explored the implications of energy vulnerability, as well as energy uncertainties, geopolitical risks, energy efficiency, and digitalization on green growth in the US. Dissimilar to the steps taken in prior studies, the current investigation harnessed updated data spanning January 2000 to December 2022 for empirical analysis. Besides, the study boosts novel empirical techniques that are lacking in existing related studies. Specifically, the study employed the recently introduced wavelet quantile regression, the wavelet quantile Granger causality, and the rolling windows wavelet quantile Granger causality for its empirical estimates. This projects the study as the first to investigate the intricate relationships between these highlighted variables and green growth in the US with these novel econometric tool kits. Given the comprehensive steps taken herein, new insights with profound policy implications were rationalized. Hence, policymakers are provided with streamlined policy insights to strengthen the green growth initiatives in the US in line with the UN's SDGs 7 and 13.

The study uncovered several novel insights about green growth and the selected determinants during the investigated period. Essentially, the study discovered that energy vulnerability and other selected series had prominent heterogeneous and asymmetric impacts on green growth across the quantiles and frequencies. This evidence is noteworthy and informs policymakers to avoid a one‐size‐fits‐all policy option. Rather, policy options to influence the effects of each determinant must be considered at each quantile and frequency to curtail possible deviations from expected effects and ensure sustained green growth in the country. Herein, ensuring consistent self‐sufficiency in energy supplies is critical to protecting the economy from the vagaries of energy vulnerability. Essentially, the momentum gained during the long term should be maintained for overall environmental sustainability. Likewise, adequate supply and stable and affordable energies are recommended to mitigate the negative impasse of energy uncertainties on growth, given that energy price instability and unpredictability are the leading causes of energy uncertainties. An urgent transition to affordable and green energies is also recommended to reduce the unpleasant repercussions of energy vulnerability and uncertainties.

The short‐term negative effects of GPR were curtailed during the medium term. However, this notable achievement was short‐lived, given the turn of events (green growth‐dampening effects) in the long term. Although the long‐term negative impasse of GPR coincides with notable global events, including the COVID‐19 global pandemic, and Russian–Ukraine, and Israel–Palestine uproars, the outcome underscores notable policy shock infiltrations arising from these global risk factors. However, policymaking should be proactive and forward‐looking to insulate the local economy from the vagaries of external shocks. Hence, it behooves environmental administrators in the US to promote green growth policies that could withstand the disruptive tendencies of external shocks.

Furthermore, the notable negative and green growth‐reducing effects of energy efficiency and digitalization demand policy attention. Not to undermine their notable green growth‐enhancing effects in some quantiles of the distributions; however, their prominent short‐term negative effects highlight some inadequacies in energy efficiency and digitalization infrastructure funding and implementation. Unarguably, funding inadequacy is a primary limiting factor of energy efficiency and environmental quality‐enhancing digital infrastructure. Hence, the US must invest in these factors to ensure they contribute optimally to green growth and overall sustainable development. However, caution is critical to curtail immediate negative impacts that could arise from intense pressure on available resources. Rather, a balanced policy overview considering competing interests' merits and time‐sensitive peculiarities would engender sustainable development and consistent environmental prosperity.

### 5.1 Limitations and Future Research

The empirical evidence portrayed within this study aligns with its defined objectives. Thus, the study explored the time‐quantile and frequency implications of energy vulnerability, energy uncertainties, geopolitical risks, energy efficiency, and digitalization on green growth in the US. The study's limiting effects are attributable to its inability to explore the perspectives of other potential predictors of green growth. Hence, future research efforts are expected to explore the implications of factors like resource efficiency, governance, green finance, open trade, energy R&D technologies, etc., on green growth. Such empirical accounts are expected to complement the submissions of this current investigation.

## Conflict of Interest

The authors declare no conflict of interest.

## Supporting information



Supplemental Table 1

## Data Availability

The data that support the findings of this study are available from the corresponding author upon reasonable request.
